# The relative importance of key life domains for people with disability: findings from a cross-sectional survey of NDIS participants in Australia

**DOI:** 10.1007/s11136-025-04067-x

**Published:** 2025-09-13

**Authors:** Samia Badji, Dennis Petrie, Anthony Harris, Gang Chen

**Affiliations:** 1 Centre for Health Economics, Monash University, 900 Dandenong Road, Caulfield East, VIC 3145 Australia; 2 Melbourne School of Population and Global Health, University of Melbourne, 207 Bouverie St, Carlton, VIC 3053 Australia

**Keywords:** Disability, Life domains, Ranking, Preference shares

## Abstract

**Purpose:**

This study investigates the relative importance for people with disability of key life domains and whether this differs between young people (15–24) and adults (25 and over).

**Methods:**

A cross-sectional survey was conducted from 20 October to 31 December 2022 with National Disability Insurance Scheme (NDIS) participants asked to rank eight domains: Choice & control, Daily living, Relationships, Home, Health & wellbeing, Lifelong learning, Work, Social, Community and Civic participation. Based on a random utility framework, the data were analysed based on a ranked-ordered logit model to estimate preference shares for the order of preferences across domains. Analyses were conducted separately for the young and adult cohorts. Sensitivity analyses were conducted by relaxing the equal importance of NDIS domains in the ranking exercise based on related life domain importance rating information, which was also collected in the survey.

**Results:**

Our sample consisted of 1140 NDIS participants. While the majority ranked the domains as equally important, answers from the rating module suggested otherwise. Adjustments for these differences lead to similar results with both age cohorts ranking Health & Wellbeing, Home and Daily living as the most important domains. These were followed by Relationships, Choice & control, Social, Community & Civic participation, Lifelong learning, and Work for younger people. For older people the importance order between the Choice & control and Relationships was switched.

**Conclusion:**

Our results revealed similarity between what younger and older people perceive as important and despite often receiving a fair share of policy attention, work was seen, on average, as the least important life domain.

**Supplementary Information:**

The online version contains supplementary material available at 10.1007/s11136-025-04067-x.

## Abstract (plain language summary)

- Understanding what matters in the lives of people with disabilities is crucial for tailoring policy decisions to their needs.
- This study surveyed people with disability who are part of the National Disability Insurance Scheme, an Australian scheme which provides personalised funding for services and support based on individual needs. Participants were tasked to rank eight life domains and our study shows that Health & Wellbeing, Home, and Daily Living were considered the most important areas for both the young (15–25 years old) and adult (25 years old and over) cohorts. Although Work is often a policy focus, this domain was ranked the least important by both cohorts.

**Supplementary Information:**

The online version contains supplementary material available at 10.1007/s11136-025-04067-x.

## Background

In 2022, 21.4% of people in Australia had a disability and approximately 7.9% had a profound or severe disability [1]. Like several other OECD countries, in the last decade Australia has moved away from a government-funded service approach to a person-centred budget approach where individuals access services and supports from the private market. Eligible National Disability Insurance Scheme (NDIS) participants use their budget to access the services and support they need (e.g. personal care and support, community access and participation, therapy and rehabilitation services, and assistive technology and equipment) based on their individual needs and goals. This shift towards a person-centred approach in disability support services has also highlighted the need for a corresponding shift in the evaluation of programs to focus on those life aspects that matter most to people with disability [2].

In this paper, we focus on understanding the relative importance to people with disability of all eight life domains, as adopted by the NDIS in their Outcome’s Framework as the key outcome domains for participants aged 15 and over. These include: Choice and Control, Daily living, Relationships, Home, Health and wellbeing, Lifelong learning, Work, Social, Community and Civic participation. These are all the domains included in the NDIS Outcomes Framework which was developed in consultation with stakeholders, including people with disability and disability organisations,^1^ however the relative importance of these domains has not yet been measured.

The literature remains mixed on how to elicit the relative importance of life domains and there is limited empirical evidence on whether incorporating domain importance into summary outcomes measures would produce significantly different findings than assuming all domains are equally important [3–7]. Assuming that life domains are equally important or relying on satisfaction only is however unlikely to reflect societal preferences. Studies have shown that societal preferences for life domain usually differ, satisfaction is not a proxy for importance and there are differences in preferences across populations and in particular across age groups [8–14]. Therefore, while eliciting the preferences for life domains can be difficult, measuring the importance of these life domains could help better target resources to areas that people with disability perceive as most important.

This study conducted a multi-module online survey of people on the NDIS and asked them to complete a ranking task of life domains. We present respondents’ rankings for each domain and summarise their responses using preference shares—the predicted proportion of the population that would rank a domain first based on the full ranking of all domains. This approach contributes to the growing body of literature on what matters most in the lives of people with disability. Furthermore, by comparing the results for young people and adults, we can further shed lights on whether the ranking of life aspects is consistent across these life stages. The findings of this study contribute to the policy agenda on building a well-being economy by understanding what matters for people with disability.

## Methods

### Survey and recruitment

A cross-sectional survey was conducted between 20 October 2022 and 31 December 2022. The survey was programmed on the Qualtrics Online Survey Platform (www.qualtrics.com/au/) and the link was distributed to over 30,000 NDIS participants (aged 15 years and over) or their nominated supporter by the National Disability Insurance Agency (NDIA). Ethics approval for this study was provided by Monash University Human Research Ethics Committee (Project ID: 32843).

The survey started with a question about who the respondent is in relation to the NDIS participant, followed by separate modules, including a module on subjective wellbeing (SW module) to understand NDIS participants’ subjective wellbeing, a ranking module to understand the relative importance of all NDIS Outcomes Framework domains (detailed in  “Ranking task” section), questions about NDIS participants’ disability history and personal characteristics, and lastly basic characteristics of the proxy respondents if the survey was not completed by NDIS participants themselves.

### Ranking task

**Fig. 1:**
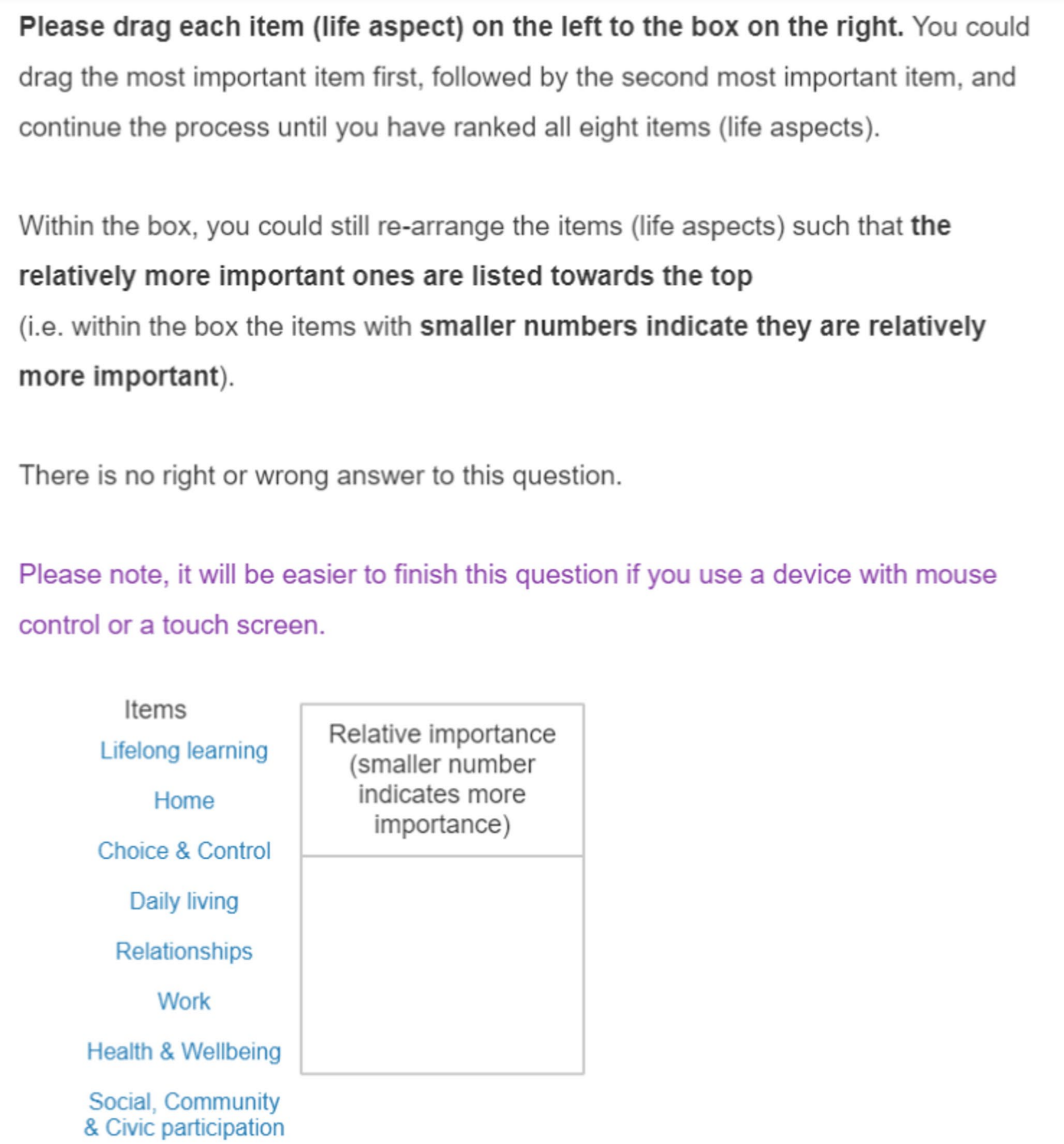
The ranking task

The ranking module presented the eight domains to the participants and comprised three possible questions (see online Appendix). The first question asked respondents whether the eight domains were equally important in their life. For those who replied no or were unsure, a second question (the ranking task) followed; see Fig. 1. In the ranking task, participants were tasked to drag each domain (presented in a randomised order on the right) into a box on the left with a higher position in the box reflecting higher importance. The third question asked participants whether some of the life domains they had dragged were equally important and if so, which ones.

### Statistical analyses

The preference of participants over a discrete set of items (the eight domains of the NDIS Outcomes Framework) is studied using the random utility framework. Let *U*_*ij*_ be the potential utility of domain *j* (*j* = 1,…,J, J = 8) for individual *i* ;

1 $$\:{U}_{ij}={V}_{ij}+{\epsilon\:}_{ij},$$

where *V* is the deterministic component of the potential utility and ε is the random component of the potential utility (i.e. the error term) assumed to have an extreme value distribution. For the deterministic component *V*, an additive functional form is assumed: $\:V={x}^{{\prime\:}}\beta\:$, where *x* is a vector that refers to the NDIS Outcomes Framework domains, and β is a vector that refers to the coefficients of interest (i.e. the potential utility an individual may derive from improvements in each domain). Let *r*_*ij*_ be the rank given to domain *j* by respondent *i*, with *r* taking any integer values from 1, the most important to 8 the least important. The ranking task allows each participant to provide a full rank of all eight domains. In such case, we know that:

2 $$\:{U}_{i{r}_{i1}}>{U}_{i{r}_{i2}}>...>{U}_{i{r}_{i8}}.$$

The ranking data are analysed by using a ranked-ordered logit model, which can be seen as a series of multinomial logit models. The coefficients of the ranked-ordered logit model are themselves difficult to directly interpret and thus we use this estimated equation to predict preference shares (e.g. the expected share of the population that would have ranked that domain as the most important).

The preference share $\:{p}_{j}\:$for domain *j* is predicted based on the estimated β coefficients using Eq. (3), which calculates the expected probability that domain *j* is most preferred by the participants.

3 $$\:{p}_{j}=\text{e}\text{x}\text{p}\left({V}_{j}\right)/{\sum\:}_{j=1}^{J}\text{e}\text{x}\text{p}\left({V}_{j}\right)$$

This is a convenient approach to summarise the preference for each domain across the sample with ranking information which not only considers what was listed as the most important domain but also where each domain was ranked across the eight domains.^2^

For the ranking data, if participants completed the task (i.e. they ranked all eight domains), the rank variable will be recorded from 1 (indicating the most important domain) to 8 (indicating the least important domain). In this study, we provided participants with an opportunity to specify equal ranking (ties) for some domains and consequently, we incorporate this information in the regression analysis. For example, if a participant indicates that the first three ranked domains should be equally important, the revised ranking information will be (1,1,1,2,3,4,5,6) instead of the raw record (1,2,3,4,5,6,7,8). For those who did not rank all eight domains, we assumed that all unranked domains were equally important though not as important as domains that were ranked and therefore unranked domains were given the next available level of ranking. If participants specified that all eight domains are ranked equally after completing the ranking task, they were grouped into the equally ranked sample and not included in the regression analysis. Therefore, the results of the regression are not sufficient to fully understand the *overall* preference share $\:{P}_{j}$ of domain j which is computed as:

4 $$\:{P}_{j}=\stackrel{-}{p}*\alpha\:+{p}_{j}*(1-\alpha\:)$$

where α is the share of participants perceiving all domains as equally important and $\:\stackrel{-}{p}\:$their preference share for all domains ($\:\stackrel{-}{p}=\frac{1}{8}=0.125$).

We analyse results separately for young people (aged 15–24) and adults (aged 25 and over), using an age cut-off consistent with the NDIS Outcomes Framework’s lifespan approach, and consider pooled analysis to further explore whether the ranking of life aspects is consistent across these life stages. The statistical analyses are conducted using Stata 17 software (StataCorp. 2019) and the ties in the ranking data are handled using Efron’s method [15].

### Sensitivity analysis

Our ranking module started with a question about whether all domains were equally important. It is possible that some respondents realised they if they answered “yes”, they could skip the ranking task (though the question only appeared on the next page). Therefore, those for whom all domains were relatively equal or the ranking task too cognitively burdensome could answer “yes” even though they may not perceive all domains equal. We therefore conducted sensitivity analyses by cross-checking those who specified equal rankings for the eight domains with their answers to items selected from the SW module based on how closely they related to the eight domains.^3^

The SW module consisted of two components. The first component has 35 items – with many that can be associated with the life domains of the NDIS Outcomes Framework – to measure the level of wellbeing. The second component is to measure the importance of the 35 items on a 5-level scale: “Not important” (1), “Slightly important” (2), “Moderately important” (3), “Very important” (4), and “Extremely important” (5). This module was used to develop a Disability Wellbeing Index [16], an instrument to measure the wellbeing of people with disability, and was also available in Easy English.

Although all eight domains from the ranking module were assigned corresponding items to the SW module, the comparison is not straightforward and the following points should be kept in mind when interpreting the results. First, the NDIS framework asks about the importance of domains in participants’ lives, while the SW module asks about the importance for overall wellbeing. Second, for the NDIS framework, the domains are ranked while in the SW module, the items are rated.^4^ Third, even when a closely related item is identified, the phrasing difference (e.g. “Work” vs. “paid work”) can impact how people perceive their importance. Moreover, an item may not fully cover all aspects of its corresponding domain. Lastly, when selecting the closest item(s) to each domain in the NDIS Outcomes Framework, the researchers’ choice for those items may not match what NDIS participants would have selected themselves.

When there seemed to be discrepancies between the ranking of domains and the rating of corresponding items (e.g. if a participant declares an item “not important” and another “very important”, we considered unlikely that their corresponding domain could be equally ranked), the sensitivity analysis provided an alternative rank of 2 for some of the domains (all originally ranked 1) depending on the ratings of their corresponding items.

An additional regression analysis was conducted for those with discrepancies (approximately half of adults and a third of young people had their originally equal ranking imputed) and their corresponding preference share computed (Version 2 hereafter).

## Results

### Descriptive analyses

Table 1 presents the characteristics of the young cohort (*N* = 228) and adult cohort (*N* = 912).^5^ In the young cohort, more than half of responses were based solely on the proxy’s understanding of their lives while a third were reported by the participant or a proxy with the participant.^6^ The young cohort is about 19 years old and slightly more male (55%). They have various types of disabilities, including intellectual (69%), sensory (56%), psychosocial (56%), autism (52%), physical (44%), head injury, stroke, acquired brain injury (9%) or other types not classified elsewhere (10%). The great majority (86%) live with their family and around 6% live in supported accommodation. Most NDIS participants report good mental and physical health.

**Table 1:** Respondent characteristics

	Young people (*N* = 228)	Adults (*N* = 912)	Equality test
	Mean/%	Mean/%	*p*-value
*Panel A—Types of responses, %*			0.614
Self-reported/Proxy with participants	34.21	42.98	
Proxy-based on understanding	60.53	52.96	
Proxy (unknown)	5.26	4.06	
*Panel B—NDIS participants’ characteristics*			
Age, mean (SD)	18.81 (2.91)	47.64 (13.64)	< 0.001
Sex, %			0.007
Male	55.70	53.73	
Female	40.35	45.39	
Another term	2.19	0.44	
Prefer not to say/missing	1.75	0.44	
*Disability types, %*			
Senses	56.14	43.64	0.001
Intellectual	68.86	54.50	< 0.001
Physical	43.86	53.95	0.008
Psychosocial	55.70	44.19	0.002
Head Injury, Stroke, Acquired Brain Injury	8.77	17.76	0.001
Autism	52.19	22.81	< 0.001
Others	10.09	12.83	0.310
Missing	0.44	0.22	0.488
*Disability acquired, %*			< 0.001
Born with or disability before 5 years old	85.96	58.33	
Less than 5 years	3.51	6.03	
6–10 years	3.95	9.32	
> 10 years	5.26	25.22	
Unsure/Missing	1.32	1.10	
*Disability support pension (DSP) status,%*			< 0.001
Yes	62.28	80.44	
No, but I used to	1.32	4.62	
No, never	36.40	14.95	
*Living situation, %*			< 0.001
Supported accomodation-self	4.82	6.80	
Supported accomodation-with other	1.75	18.86	
Home-alone	2.19	13.16	
Live with family	85.53	52.08	
Live with support worker/carer (not family)	0.44	1.32	
Others	5.26	7.46	
Missing	0.00	0.33	
*Self-reported physical health*			
Excellent	6.14	2.85	
Very good	18.86	13.27	
Good	34.65	25.77	
Fair	26.32	31.25	
Poor	13.60	26.86	
Missing	0.44	0.00	
*Self-reported mental health*			0.664
Excellent	3.07	3.4	
Very good	15.79	15.57	
Good	31.58	26.75	
Fair	28.95	31.03	
Poor	20.18	22.92	
Missing	0.44	0.33	

One individual can have several disability types. Student t-test with unequal variance is used for age, and Fisher’s exact tests for other variables. Missing categories are excluded from the tests

The adult cohort is about 48 years old and slightly more male (54%). Their proxy responded with them or they answered themselves for 43% of the sample while for 53% the proxy responded based on their understanding of the life of the NDIS participant. Their most reported types of disability are intellectual (55%), physical (54%), sensory (44%), psychosocial (44%), autism (23%) and head injury, stroke, acquired brain injury (18%). The majority (58%) acquired their disability before the age of 5. Most respondents (80%) currently receive disability support pension. The majority live with their families (52%). Some live in supported accommodation with others (19%) or alone (7%), while many live alone at home (13%). For both mental and physical health, approximately half of adults consider their health as at least good while approximately a quarter consider it poor.

Among 1140 participants, the ranking information of 46 participants was missing and they were excluded. Of the remaining 1,083 participants, 404 participants complete the ranking task whilst 679 participants specified that all eight domains were equally important. In the sensitivity analysis, 335 of these 679 participants had their rankings amended based on the two-level ranking variable created due to possible discrepancies with the importance ratings they gave to items relating to these domains in the SW module.

**Fig. 2:**
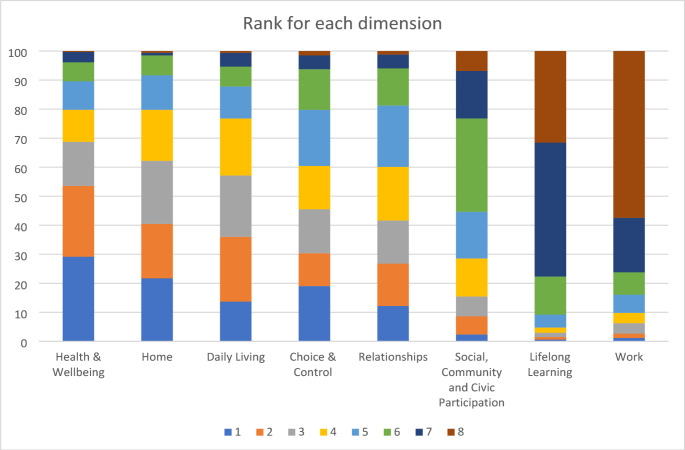
Distribution of ranking given for each of the eight life domains (1-the most important domain, 8-the least important domain) by Young people and Adults

Figure 2 shows the distributions of raw ranking information among those who completed the ranking exercise. It highlights that the Health & Wellbeing domain is the most likely to be ranked first (most important) whilst Work is most likely to be ranked last.

### Ranking data analyses

The regression results are reported in Table 2. The work domain is the reference group and the magnitudes of the coefficients represent the strength of the preference against the Work domain. Using the coefficient of the Health & Wellbeing domain (β = 1.948) in Column (1) as an example, it suggests that the odds of preferring Health & Wellbeing over Work is 7 (= exp(1.948)).

**Table 2:** Ranked-ordered logit model estimates on the relative importance of NDIS outcomes framework domains

NDIS domains (Ref. Work)	(1)	(2)	(3)	(4)
	Young People^†^	Young People^‡^	Adults^†^	Adults^‡^
Health and Wellbeing	1.948	1.544	2.217	1.612
	(0.252)**	(0.147)**	(0.131)**	(0.065)**
Home	1.942	1.643	2.148	1.808
	(0.251)**	(0.136)**	(0.117)**	(0.043)**
Daily living	1.723	1.618	2.081	1.705
	(0.231)**	(0.139)**	(0.118)**	(0.057)**
Choice and Control	1.384	1.573	1.843	1.661
	(0.221)**	(0.146)**	(0.110)**	(0.061)**
Relationships	1.578	1.558	1.776	1.717
	(0.208)**	(0.151)**	(0.111)**	(0.055)**
Social, Community and Civic Participation	0.773	1.279	1.186	1.259
	(0.210)**	(0.179)**	(0.096)**	(0.080)**
Lifelong learning	0.489	1.127	0.283	0.471
	(0.194)*	(0.178)**	(0.081)**	(0.055)**
N	68	60	335	275

Robust standard errors are in parentheses. **p < 0.01, *p < 0.05. ^†^Columns (1) & (3): Participants who ranked the eight domains; ^‡^ Columns (2) & (4): Imputed values for participants who reported equal ranking on eight domains but reported unequal ratings for related items in the SW module and who will be included in the sensitivity analyses (see online Appendix for their characteristics)

Columns (1) & (3) report the results among participants who gave full rank on eight NDIS Outcomes Framework domains, whilst Columns (2) & (4) report the results based on the imputed two-level ranking data among participants who are identified from sensitivity analyses as actually unlikely to see the eight life domains as equally important. Overall, Health & Wellbeing, Home, and Daily living are the three most important domains whilst Work is the least important domain and this result is robust across analyses.^7^ While a statistical analysis supported the analysis of the two age cohorts separately, differences were minimal and results similar.^8^

### Preference share

The weighted preference share is calculated for the Young people cohort (Table 3) and Adult cohort (Table 4) separately using the regression coefficients reported in Table 2. Version 1 is calculated as the weighted average of two groups of participants, those who specify equal importance of eight domains and those who provided full ranking on eight domains. Version 2 further separates the former group into two sub-groups as the sensitivity analyses.

**Table 3:** Preference share of NDIS outcomes framework domains, young people cohort

NDIS domains	Logit based $\:{p}_{j}$^†^	Equal importance $\:\stackrel{-}{p}$	Weighted average ($\:{P}_{j})$
*Panel A—Preference share (version 1, V1)*			
Health and wellbeing	0.211	0.125	0.15
Home	0.210	0.125	0.15
Daily living	0.169	0.125	0.14
Choice and control	0.120	0.125	0.12
Relationships	0.146	0.125	0.13
Social, community and civic participation	0.065	0.125	0.11
Lifelong learning	0.049	0.125	0.10
Work	0.030	0.125	0.10
% of N	30.5%	69.5%	

NDIS DOMAINS	Logit based $\:{p}_{j}$^†^	Logit based $\:{p}_{j}$^‡^	Equal Importance $\:\stackrel{-}{p}$	Weighted Average ($\:{P}_{j})$
*Panel B—Preference share (version 2, V2)*				
Health & Wellbeing	0.211	0.146	0.125	0.16
Home	0.210	0.161	0.125	0.16
Daily living	0.169	0.157	0.125	0.15
Choice & Control	0.120	0.150	0.125	0.13
Relationships	0.146	0.148	0.125	0.14
Social, Community & Civic participation	0.065	0.112	0.125	0.10
Lifelong learning	0.049	0.096	0.125	0.09
Work	0.030	0.031	0.125	0.07
% of N	30.5%	28.3%	41.3%	

^†^Participants who ranked the eight domains; ^‡^Participants identified as misreporting equal ranking of the eight life domains with imputed ranking values based on the sensitivity analyses (see online Appendix for their characteristics). Preference shares computed based on the formula in “Statistical analyses” section and the results of Table 2

**Table 4:** Preference share of NDIS outcomes framework domains, adults cohort

NDIS domains	Logit based $\:{p}_{j}$^†^	Equal Importance $\:\stackrel{-}{p}$	Weighted Average ($\:{P}_{j})$
*Panel A—Preference share (Version 1, V1)*			
Health and wellbeing	0.210	0.125	0.16
Home	0.198	0.125	0.15
Daily living	0.183	0.125	0.15
Choice and control	0.145	0.125	0.13
Relationships	0.136	0.125	0.13
Social, community and civic participation	0.075	0.125	0.11
Lifelong learning	0.030	0.125	0.09
Work	0.023	0.125	0.09
% of N	38.8%	61.2%	

NDIS domains	Logit based $\:{p}_{j}$^†^	Logit based $\:{p}_{j}$^‡^	Equal Importance $\:\stackrel{-}{p}$	Weighted Average ($\:{P}_{j})$
Health and Wellbeing	0.210	0.149	0.125	0.17
Home	0.198	0.182	0.125	0.17
Daily living	0.183	0.164	0.125	0.16
Choice and control	0.145	0.157	0.125	0.14
Relationships	0.136	0.166	0.125	0.14
Social, community and civic participation	0.075	0.105	0.125	0.10
Lifelong learning	0.030	0.048	0.125	0.06
Work	0.023	0.030	0.125	0.05
% of N	38.8%	32.2%	29.0%	

^†^Participants who ranked the eight domains; ‡ Participants identified as misreporting equal ranking of the eight life domains with imputed ranking values based on the sensitivity analyses (see online Appendix for their characteristics). Preference shares computed based on the formula in “Statistical analyses” section and the results of Table 2

Generally speaking, the findings of weighted average preference share are consistent with the regression estimates that Health & Wellbeing, Home, and Daily living are the three most important domains whilst Work is the least important domain. Comparing V1 and V2 results, we see that the weighted preference share drops for Work and slightly drops for Lifelong learning, and Social, Community & Civic participation once we account for potential discrepancies (V2) while all remaining domains have their corresponding share slightly increase.

## Discussion

This study elicited NDIS participants’ views about the relative importance of the eight life domains in the NDIS Outcomes Framework for the young and adult cohorts. The findings indicate that for both cohorts, the following three domains are in general considered the most important: Health & Wellbeing, Home, and Daily living while the three domains considered least important were Work, Lifelong learning, and Social, Community & Civic Participation each contributing to less than 12.5% (i.e. the expected preference share if all domains were equally important).

The finding that Health & Wellbeing was, in general, considered the most important domain is consistent with what has been reported in the stated preference literature that focuses on broader wellbeing domains in Australia [17]. A study on men with spinal cord injury had the item Health and Personal safety as the most important while the Work item was somewhere in the middle [18]. A systematic review of the health and life priorities of people with spinal cord injuries found that Health and Relationships were the most important life areas in the majority of studies reviewed. Work was not in the top 25% of items listed although it was important in many of those studies [19].

Our results show that Work is considered, in general, as the least important domain. It should be noted that the impairment of people with severe disability interacting with societal barriers may limit the opportunity for them to engage in work-related activities; hence some participants may perceive work as not relevant to them and consequently rank Work relatively low as compared to other domains. This is consistent with another study where men with tetraplegia had an 8% points lower probability to report Work as “important” or “very important” compared to men with paraplegia [18]. By using the responses to the SW module, we analysed a subsample (N = 256) of NDIS adult participants who gave a rating for their satisfaction related to Work (that is who did not specify ”unsure”, ”not relevant” or left the Work item unanswered). The results indicate that in this subsample, Lifelong learning becomes the least important domain whilst Work become the second least important domain, which is also statistically indifferent from the third least important domain, Social, Community & Civic participation. We conducted additional analyses to rank the eight domains based solely on the SW module items’ importance ratings, ignoring the ranking answers and found again that the least important domain was Work, followed by Lifelong learning and Social, Community & Civic Participation. Given that the NDIS is generally comprised of people with severe disability, and our sample has a large number of proxy respondents many of whom may have a particularly severe disability, it is not surprising that respondents do not rank Work as an important domain *relative* to the other domains. It is possible that with a more inclusive society, more possibilities open up for people with disabilities to work such that the Work domain could potentially become more important to them over time.

Our results can be compared with Crocker et al. [2]’s Australian study that included people with disability and tasked participants to rank 12 items by importance to their quality of life and wellbeing (sleep, independence, physical mobility, mental wellbeing, control, self-care, pain, vision, hearing, safety, social-relationships, dignity). They found that people with disability ranked Control and Indepence in the top 2 (similar to our “Choice and Control” ranked 4th for adults), self care was ranked third (similar to “daily living” also ranked 3rd in our study), Mental wellbeing was ranked 4th (similar to “Health & wellbeing”). Social relationships in Crocket et al.’s study can be seen as similar to Relationships or Social, Community & Civic Participation in our survey and both are ranked in the top of the bottom half. The comparison, however, is difficult as several items are not directly relevant to the NDIS framework (such as sleep, dignity and safety) or missing (Work). Other items are very specific functionning items (vision and hearing) and while some may group such items into `health’ we are wary of considering a blind or deaf person to never have the best possible physical health. It is possible that the concept of physical health for those gradually losing sight or hearing may fundamentally differ from those born deaf or blind and it is possible that many people with disabilities identify with a concept of physical health that differ to that of functioning. If `vision’ and `hearing’ were seen as part of the health domain, Crocker et al. (2021)’s results would be in contradiction with ours. We showed Health and Wellbeing as the most important aspects in the life of people with disabilities whereas vision and hearing were seen as the least important in Crocker et al. [2]’s study.

There are some caveats to our study. We collected our responses from an online survey. Due to our target population of NDIS participants who are by definition a subgroup of people with significant disability caused by a permanent impairment, it can be very difficult or even impossible to obtain responses directly from all NDIS participants themselves. The ranking questions were answered by NDIS participants themselves, proxy respondents who completed the survey together with the NDIS participants and proxy respondents who completed the survey based on their understanding of the life of the NDIS participants. Although there are differences in the characteristics of NDIS participants by reporting types which could explain differences in the ranking of life dimensions, it is also well-recognised that individuals and proxy respondents tend to give different answers [20–22]. It is worth noting that some have found that when the reports are averaged at the group level, the approximation of the proxy report becomes very close to that of the individuals themselves [23]. Moreover, in the absence of differences that are specific to some life domains, the ranking should be similar. Roydhouse, Gutman, Keating, Mor, and Wilson [24] report similar proxy-patient difference sizes regardless of the health domain. Due to the possibility of differences solely due to the proxy responding based on their understanding. Future studies should strive to increase the level of self-report, potentially by using other methods such as interviews [25].

A key limitation of this study is the low response rate. With over 30,000 people who received a link to the survey, around 1,600 clicked the survey link and completed some part of the survey. In this study, we focused on a subsample of 1,140 respondents who went through all NDIS participant-related sections of the online survey. We therefore cannot rule out that survey respondents are a selective sample. Meanwhile, it is encouraging to see that the respondents to the survey cover people with different types of disabilities. Future studies should consider providing a financial reimbursement to the respondents for their time and effort, which should help increase the survey response rate. It should also be remembered that the survey was administered online, and may not have been easily accessible to participants with some types of disability. Future studies could explore multiple modes for data collection to better meet the needs of people with disability. Last but not least the survey length could influence the survey completion rate. The ranking task was part of a larger survey to understand the wellbeing of people with disability. A shorter more focused survey would reduce the potential response burden and improve participants’ engagement.

The length of the survey could also explain why around half of the respondents stated that all of the eight domains from the NDIS Outcomes Framework were equal although their rating of related items suggested otherwise. The complexity in finding the right item(s) for each of the eight NDIS Outcomes Framework domains and the difficulty in aggregating items within the domain could also explain why there appear to be many inconsistent answers. Despite these potential discrepancies at the individual level, the overall ranking of the eight life domains across the population remained very consistent even after accounting for these individual discrepancies in the sensitivity analysis. The sensitivity analysis had several limitations due to differences in what domains were supposed to matter for (life vs. subjective wellbeing), how importance was assessed (ranking vs. rating) and the phrasing. However, all these limitations are unlikely to systematically favour one life domain over another one and therefore the sensitivity analysis remains useful to shed light on the perceived importance of those life domains.

## Conclusion

The results provide a better understanding of the priorities and needs of NDIS participants, which can inform the development of more effective interventions and policies. Given the importance of the NDIS Outcomes Framework for assessing the effectiveness of programs and tracking improvement, it is crucial to account for the relative importance of each domain carefully as shared by NDIS participants to better understand the overall impact of those programs on the broader benefits of NDIS participants. Those results can help inform policy more broadly that affect people with severe and permanent disability by encompassing better the perspective of people with disability.

## Supplementary Information

Below is the link to the electronic supplementary material.

Supplementary Material 1

## Footnotes

1 The National Disability Insurance Agency (NDIA) developed and adopted this Framework, which categorizes individuals aged 15 and over into two groups—those aged 15–24 and those aged 25 and above—to reflect their differing life stages. It was designed to measure the medium to long-term benefits of the NDIS based on a review of national and international frameworks, a review of available data for benchmarking, extensive consultation and process involving people with disability, disability researchers, experts working with specific communities, peak bodies with a pilot involving almost 400 interviews conducted. For more information, see https://www.ndis.gov.au/publications/outcomes-framework-pilot-study-summary-report-2015 (Access date: 23/07/2025).

2 The coefficient for the reference domain (i.e. Work) is zero.

3 The online Appendix provides more details and provides the full details of the SW module. For completeness, the appendix also provides the ranking if the analysis was *solely* based on answers to the SW module.

4 Therefore, it is not clear to what extent, a participant would consider different levels (such as “Very important” and “Extremely important”) as somewhat equally important nor whether items with identical rating (e.g. “Very important”) would be seen to have identical importance in a ranking task (and therefore identical rank).

5 The online Appendix presents the characteristics of respondents by reporting type (proxy only or not). It also compares this sample to individuals who began but did not complete the survey, in terms of respondent type and survey version used.

6 Other responses were from a proxy but it is unclear whether the NDIS participant was answering together with the proxy.

7 Using *only* the SW module (ratings) similarly identified Work as the least important domain (see online Appendix E).

8 Regressions incorporating interaction terms between domains and the age-cohort dummy rejected the null hypothesis that all interaction terms were zero in both Version 1 and Version 2. However, the specific nature of these differences remains unclear as only the Lifelong learning domain showed a statistically significant interaction at the 5% level, and only in Version 2. See online Appendix FF for further details.
