# Supplementary material for: The relative importance of key life domains for people with disability: findings from a cross-sectional survey of NDIS participants in Australia
Source: Qual Life Res. 2025 Sep 13;34(11):3121–31. doi: 10.1007/s11136-025-04067-x (PMC12681490; doi:10.1007/s11136-025-04067-x)
Supplement: Supplementary file 1 — Supplementary Material 1 [file 11136_2025_4067_MOESM1_ESM.docx]

## **Supplementary Information**

**The Relative Importance of Key Life Domains for People with Disability:**

**Findings from a cross-sectional survey of NDIS participants in Australia**

**Quality of Life Research**

Samia Badji^1^, Dennis Petrie^1^, Anthony Harris^1^, Gang Chen^1,2^

^1^ Centre for Health Economics, Monash University

900 Dandenong Road
Caulfield East, VIC 3145
Australia

^2^ Melbourne School of Population and Global Health, University of Melbourne

207 Bouverie St

Carlton VIC 3053

Australia

[Samia.badji@monash.edu](mailto:Samia.badji@monash.edu) (corresponding author) <https://orcid.org/0000-0001-7352-0232>

## **Appendix A. Section C of the online survey (Ranking): Eliciting the relative importance of each of the eight life domains**


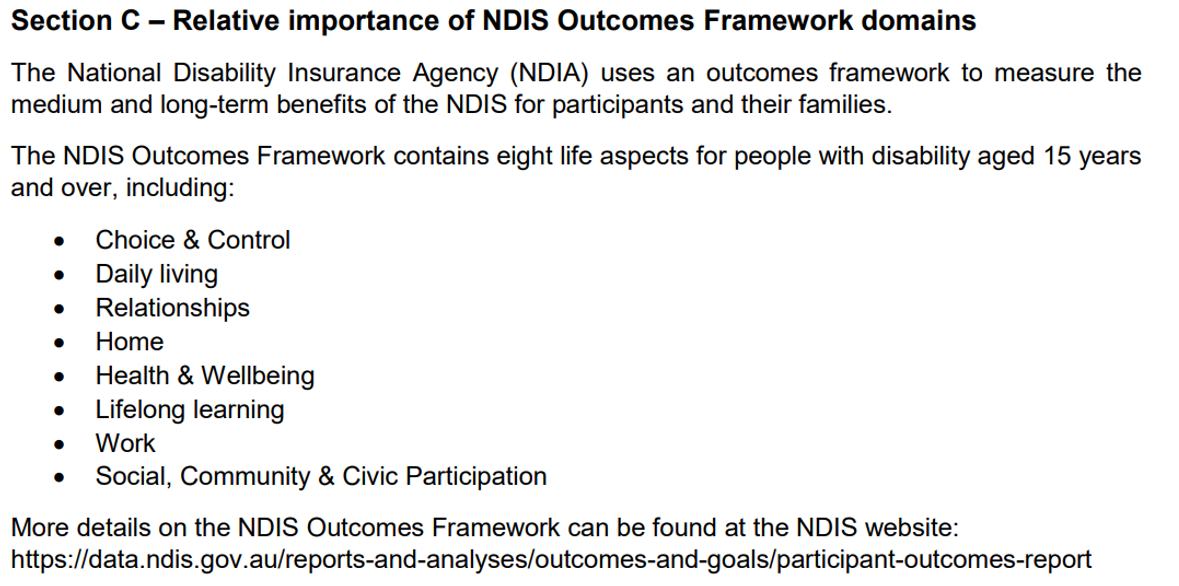


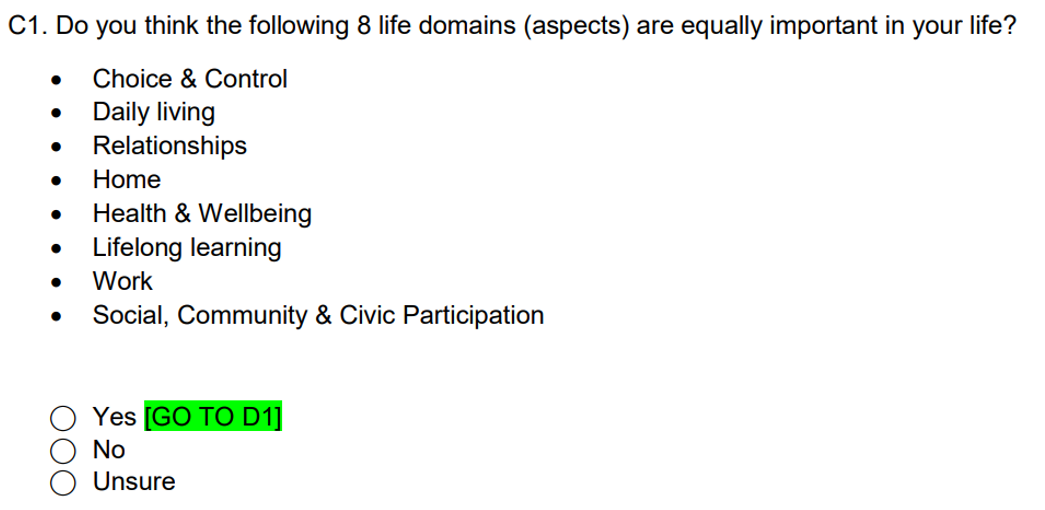


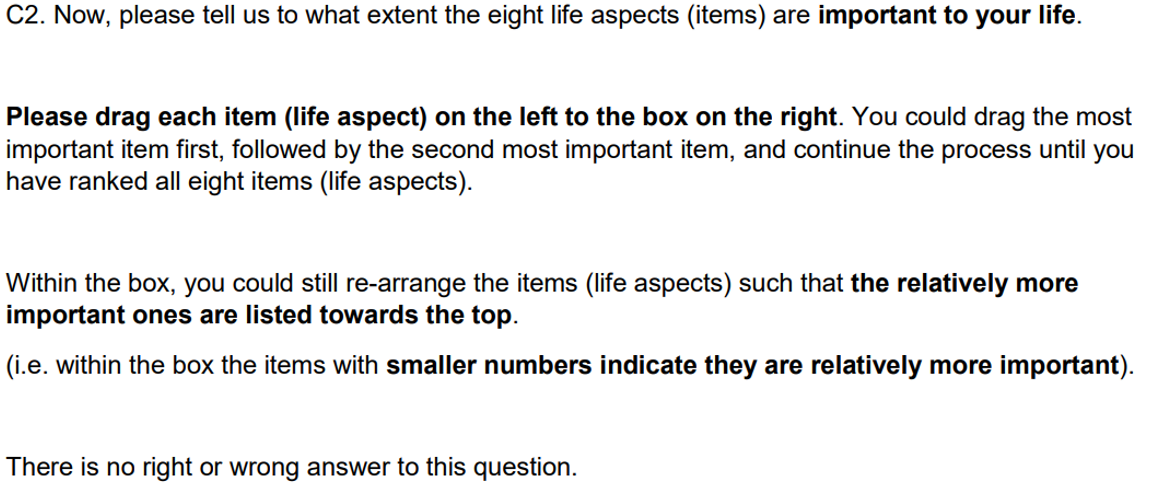


C3.


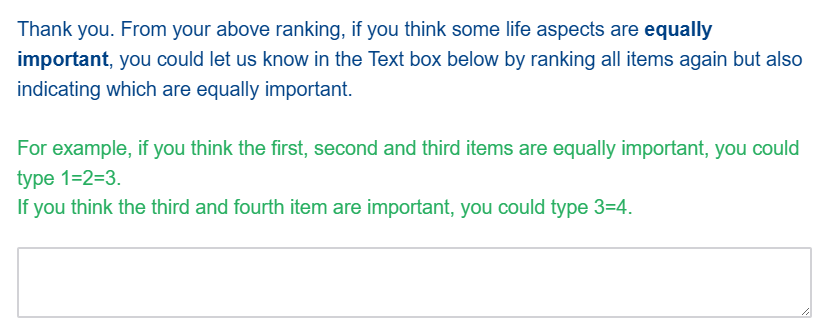
**Appendix B. The domains and items included in the Subjective wellbeing module (with a rating task)**

**1. Relationships (5 items)**

I am satisfied with the way I get along with people

(for example, friends, family, carers, support workers)

I am satisfied with the way I get along with my family

I am satisfied with my friendships

I have the opportunity for intimate relationships

I am satisfied with the way I get along with people who support me

(for example, support workers, allied health, counsellors)

**2. Daily life (2 items)**

I am satisfied with my personal care

(for example, eating, washing, dressing, with or without the help of others)

I am satisfied with my day-to-day activities

(for example, cooking, shopping, sorting out problems, with or without the help of others)

**3. Community (3 items)**

I take part in my community as much as I want to

(for example, doing sports, going to music and cultural events, religious groups)

I feel accepted and included in my communities, in person or online

I do things in my life that are important to me

**4. Leisure time (1 item)**

I spend my leisure time doing things I enjoy

(for example, hobbies, movies, holidays) *[Example shown to the adult cohort]*

(for example, reading, social media, sport, hobbies, online streaming) *[Example shown to the young cohort]*

**5. Health (3 items)**

I am satisfied with my physical health

I am satisfied with my mental health

I am satisfied with the way my pain is managed

**6. Housing (3 items)**

I am satisfied with my living situation

I am satisfied with where I live

I am satisfied with who I live with

**7. Work (4 items)**

I am satisfied with the work I do

(for example, paid employment, unpaid caring, volunteering)

I am satisfied with the paid work I do

I am satisfied with the unpaid activities that I do for others

I am satisfied with the opportunity to follow my chosen career

**8. Learning (2 items)**

I learn new things as much as I want to

I am satisfied with my opportunities for education

(for example, at school, vocational training, university)

**9. Respect and Dignity (5 items)**

I am satisfied with how people treat me

I am satisfied with how people treat me at home

I am satisfied with how people treat me in the community

I am satisfied with how people treat me when I get services

(for example, health services, education, employment, community services)

I am able to speak up about the things that are important to me

**10. Choices (1 item)**

I control how I live my life

(for example, where I go, what I do, what I eat, who I have as friends, who my services providers are)

**11. Safety (3 items)**

I feel safe in my life

I feel safe in my home

I feel safe when I am out in the community

**12. Finances (2 items)**

I have enough money to do things that are important to me

(for example, going out with friends, buying food, buying medication)

I have control over my money to do the things I want to do

**13. Support (1 item)**

I am satisfied with the support I get

(for example, services, unpaid support)

## **Appendix C. Link between NDIS Outcome Framework domains and SW module domains**


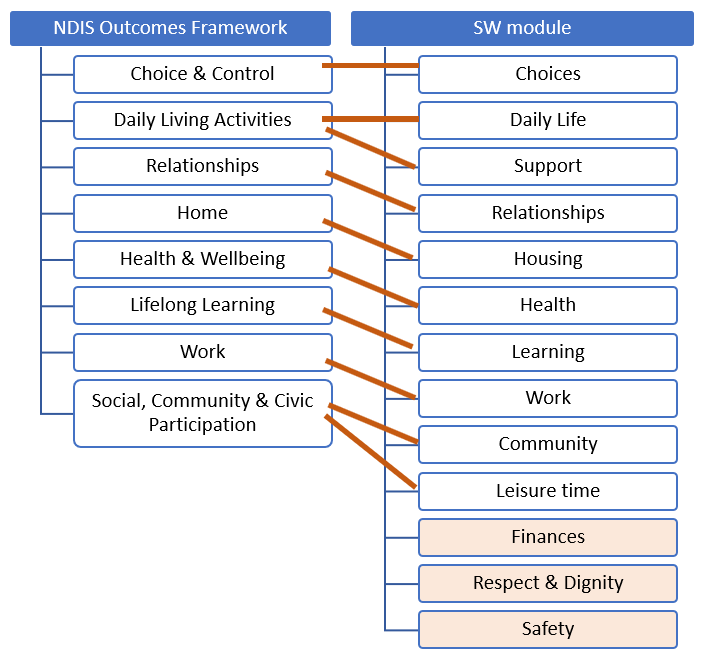


Figure S1. Relationship between the subjective wellbeing domains (from the 35-item DWI pilot) to the NDIS Outcomes Framework domains

## **Appendix D. Characteristics of respondents by reporting type**

Table S1. Respondent characteristics by types of reporting (self-reported and together with proxy vs proxy based on understanding)

|  | Self-reported or together with proxy (N=470) | Proxy based on understanding of life of NDIS participant  (N=621) | Equality test |
| --- | --- | --- | --- |
|  | Mean/% | Mean/% | p-value |
| **Age**, mean (SD) | 44.11 (16.74) | 40.16 (16.53) | <0.001 |
| **Sex**, % |  |  | 0.001 |
| Male | 47.02 | 58.62 |  |
| Female | 51.06 | 40.10 |  |
| Another term | 1.06 | 0.64 |  |
| Prefer not to say/missing | 0.85 | 0.64 |  |
| **Disability types**, % |  |  |  |
| Senses | 40.43 | 50.56 | 0.001 |
| Intellectual | 36.81 | 72.95 | <0.001 |
| Physical | 58.09 | 47.50 | 0.001 |
| Psychosocial | 41.91 | 50.08 | 0.008 |
| Head Injury, Stroke, Acquired Brain injury | 14.89 | 16.91 | 0.405 |
| Autism | 22.98 | 33.17 | <0.001 |
| Others | 12.98 | 11.76 | 0.577 |
| Missing | 0.21 | 0.00 | 0.431 |
| **Disability Acquired**, % |  |  | <0.001 |
| Born with or disability before 5 years old | 50.64 | 74.40 |  |
| Less than 5 years | 6.81 | 4.51 |  |
| 6-10 years | 11.70 | 5.96 |  |
| >10 years | 29.79 | 14.81 |  |
| Unsure/Missing | 1.06 | 0.32 |  |
| **Disability Support Pension (DSP) status, %** |  |  | <0.001 |
| Yes | 71.06 | 81.61 |  |
| No, but I used to | 5.53 | 2.90 |  |
| No, never | 23.40 | 15.48 |  |
| **Living situation, %** |  |  | <0.001 |
| Supported accommodation-self | 4.47 | 8.05 |  |
| Supported accommodation-with other | 5.53 | 23.03 |  |
| Home-alone | 13.62 | 8.53 |  |
| Live with family | 68.3 | 51.85 |  |
| Live with support worker/carer(not family) | 0.85 | 1.45 |  |
| Others | 7.02 | 6.92 |  |
| Missing | 0.21 | 0.16 |  |
| **Self-reported physical health** |  |  | 0.006 |
| Excellent | 2.55 | 4.35 |  |
| Very good | 11.49 | 16.75 |  |
| Good | 27.23 | 27.86 |  |
| Fair | 29.79 | 29.79 |  |
| Poor | 28.94 | 21.10 |  |
| Missing | 0.00 | 0.16 |  |
| **Self-reported mental health** |  |  | 0.058 |
| Excellent | 4.26 | 2.42 |  |
| Very good | 17.02 | 14.98 |  |
| Good | 23.40 | 30.43 |  |
| Fair | 32.55 | 29.79 |  |
| Poor | 22.55 | 21.90 |  |
| Missing | 0.21 | 0.48 |  |

*Notes:* One individual can have several disability types. Student t test with unequal variance is used for age, Fisher’s exact tests for other variables. Missing category excluded from the tests.

The total sample size does not match the total sample size in Table 1 as this table excluded respondents from the proxy for whom we do not know whether they responded to the survey with the NDIS participant or based on their own understanding.

Table S2. Characteristics of respondents who answered the main questions of the survey versus those who only answered the first questions of the survey (among those who gave consent)

|  | Survey sample  not part of main sample (41.8%) | Main Sample  (58.2%) |
| --- | --- | --- |
| A. Who is responding |  |  |
| NDIS participant | 8.1% | 19.2% |
| Spouse/partner | 10.8% | 10.3% |
| Parent | 55.5% | 52.3% |
| Brother/sister | 12.9% | 11.9% |
| Another family member | 6.1% | 3.2% |
| I am someone else | 6.6% | 3.1% |
| B. Standard English (vs Easy English) | 85.1% | 94.8% |

*Notes:* The differences between question A and question B are statistically significant at 5%. The survey sample includes all those who decided to respond to the survey and gave consent but are not part of our main sample (therefore they started but did not complete the survey). Other demographic characteristics are only available at the end of the survey and therefore not available to most of the survey sample that is not part of our main sample

## **Appendix E. Adjusting for potential discrepancies**

The ranking of all eight life domains may have been more cognitively burdensome to some individuals who may have observed that responding that all domains were equal meant the ranking question was skipped. For this reason, for those who responded that all domains were equal, we also analysed their answers to the subjective wellbeing (SW) module to understand if their rating could shed light on the ranking of the NDIS domains.

**Which SW items were selected to represent each NDIS domain?**

All the SW items are presented in Appendix B. We used the conceptual mapping of the two instruments (Figure S1) to assign SW items to each of the eight domains as shown in Table S3. For an individual with the same preferences, the higher the number of items within a domain, the higher the number of possible average values for that domain and therefore the lower the likelihood of computing the same ratings across domains. To mitigate this issue, where possible, we chose to assign less rather than more items for each domain.

Where only one item was available (e.g., the domain Choice & Control), we simply selected that corresponding item. For the Work domain, we retained the Paid Work item. In domains where one item more broadly captured the domain than others, we selected that general item (e.g., Relationships and Home). Selecting which of the two items for Lifelong learning best represented the domain was not straightforward, so both were retained. For Social, Community & Civic participation, we felt that Inclusion and Important Things were less related to participation. The Leisure Time item was also less about social and community participation, so only the Community item on taking part in the community was included. For Daily living, both items under Daily Life were selected, while the item in the Support domain was deemed less directly relevant. Similarly, for Health & Wellbeing, both Physical Health and Mental Health items were included, but Pain was considered less clearly associated and was excluded

Table S3. Matching the NDIS Outcome Framework domains with representative DWI items

| **NDIS Outcomes Framework Domains** | **Subjective wellbeing items** | |
| --- | --- | --- |
| Health & Wellbeing | I am satisfied with my physical health | |
|  | I am satisfied with my mental health  I am satisfied with the way my pain is managed | |
| Home | I am satisfied with my living situation  I am satisfied with where I live  I am satisfied with who I live with | |
| Daily living | (Daily Life)I am satisfied with my personal care (for example, eating, washing, dressing, with or without the help of others) | |
|  | (Daily Life) I am satisfied with my day-to-day activities (for example, cooking, shopping, sorting out problems, with or without the help of others) | |
|  | (Support) I am satisfied with the support I get (for example, services, unpaid support) | |
| Choice & Control | I control how I live my life (for example, where I go, what I do, what I eat, who I have as friends, who my service providers are) | |
| Relationships | I am satisfied with the way I get along with people (for example, friends, family, carers, support workers)  I am satisfied with the way I get along with my family  I am satisfied with my friendships  I have the opportunity for intimate relationships  I am satisfied with the way I get along with people who support me (for example, support workers, allied health, counsellors) | |
| Social, Community & Civic participation | (Community) I take part in my community as much as I want to (for example, doing sports, going to music and cultural events, religious groups) | |
|  | (Community) I feel accepted and included in my communities, in person or online | |
|  | (Community) I do things in my life that are important to me | |
|  | (Leisure Time) I spend my leisure time doing things I enjoy  (for example, hobbies, movies, holidays) *[ adult cohort]*  (for example, reading, social media, sport, hobbies, online streaming) *[ young cohort]* | |
| Lifelong learning | I learn new things as much as I want to | |
|  | I am satisfied with my opportunities for education (for example, at school, vocational training, university) | |
| Work | | I am satisfied with the paid work I do  I am satisfied with the work I do (for example, paid employment, unpaid caring, volunteering)  I am satisfied with the unpaid activities that I do for others  I am satisfied with the opportunity to follow my chosen career |

*Notes:* Where a domain from the NDIS Outcomes Framework corresponded to more than one domain in the subjective wellbeing module, we name in brackets the SW domains for those items. Items in grey have not been used to rank the NDIS domain.

#### **How to define equal importance of the NDIS domains based on the ratings in the SW module?**

To cross-check whether those who reported that the eight life domains were equally important, we use the responses to the SW module and apply the following rule:

- When one item is rated as “Not important”^[[1]](#footnote-2)^ while at least another item is rated as “Moderately important”, “Very important”, or “Extremely important”, we conclude that the NDIS domains are unlikely to have equal importance. Otherwise, we assume that the equal ranking of the domains is likely to be true.

Following this approach, approximately half of those who specify “equally important” on all eight domains should have answered otherwise. Their characteristics are presented in Table S4.

#### **How to Create an alternative ranking variable (Version 2 or “sensitivity analysis”) based on item rating?**

Following the cross-checking procedure, a new two-level ranking variable^[[2]](#footnote-3)^ was created for those participants who stated that the eight life domains were equally important but for whom we consider that this may not hold given their rating of the importance of items within that domain.

- We assigned a rank of 1 to all items that were rated as at least “Moderately important” and a rank of 2 to items rated as “Not important”.
- For items rated as “Slightly important”, we assigned a rank of 1 if they were considered more important than the median rating and a rank of 2 otherwise.

Table S4. Respondent characteristics of those with suspected incorrect ranking

|  | Young people (N=63) |  | Adults (N=281) | Equality test |
| --- | --- | --- | --- | --- |
|  | Mean/% |  | Mean/% | p-value |
| **PANEL A - Types of responses, %** |  |  |  | 0.057 |
| Self-reported/Proxy with participants | 27.0 |  | 41.6 |  |
| Proxy based on understanding | 66.7 |  | 55.2 |  |
| Proxy (unknown) | 6.4 |  | 3.2 |  |
| **PANEL B- NDIS Participants' characteristics** |  |  |  |  |
| **Age**, mean (SD) | 18.73 (2.96) |  | 48.78 (13.69) | <0.001 |
| **Sex**, % |  |  |  | 0.026 |
| Male | 68.25 |  | 53.02 |  |
| Female | 28.57 |  | 45.91 |  |
| Another term | 1.59 |  | 0.71 |  |
| Prefer not to say/missing | 1.59 |  | 0.36 |  |
| **Disability types**, % |  |  |  |  |
| Senses | 63.49 |  | 47.69 | 0.026 |
| Intellectual | 79.37 |  | 60.50 | 0.006 |
| Physical | 44.44 |  | 56.58 | 0.093 |
| Psychosocial | 63.49 |  | 49.11 | 0.050 |
| Head Injury, Stroke, Acquired Brain injury | 17.46 |  | 24.20 | 0.320 |
| Autism | 55.56 |  | 18.86 | <0.001 |
| Others | 11.11 |  | 17.44 | 0.261 |
| Missing | 0.00 |  | 0.00 | N/A |
| **Disability Acquired**, % |  |  |  | <0.001 |
| Born with or disability before 5 years old | 84.13 |  | 54.80 |  |
| Less than 5 years | 3.17 |  | 8.54 |  |
| 6-10 years | 3.17 |  | 8.90 |  |
| >10 years | 9.52 |  | 27.40 |  |
| Unsure/Missing | 0.00 |  | 0.36 |  |
| **Disability Support Pension (DSP) status,%** |  |  |  | <0.001 |
| Yes | 68.25 |  | 83.99 |  |
| No, but I used to | 1.59 |  | 5.34 |  |
| No, never | 30.16 |  | 10.68 |  |
| **Living situation, %** |  |  |  | <0.001 |
| Supported accommodation-self | 7.94 |  | 7.83 |  |
| Supported accommodation-with other | 1.59 |  | 19.57 |  |
| Home-alone | 1.59 |  | 8.19 |  |
| Live with family | 77.78 |  | 54.09 |  |
| Live with support worker/carer(not family) | 1.59 |  | 1.42 |  |
| Others | 9.52 |  | 8.54 |  |
| Missing | 0.00 |  | 0.36 |  |
| **Self-reported physical health** |  |  |  | 0.001 |
| Excellent | 6.35 |  | 2.14 |  |
| Very good | 15.87 |  | 11.03 |  |
| Good | 41.27 |  | 23.13 |  |
| Fair | 20.63 |  | 32.38 |  |
| Poor | 15.87 |  | 31.32 |  |
| Missing | 0.00 |  | 0.00 |  |
| **Self-reported mental health** |  |  |  | 0.187 |
| Excellent | 3.17 |  | 2.85 |  |
| Very good | 12.70 |  | 11.74 |  |
| Good | 41.27 |  | 29.18 |  |
| Fair | 30.16 |  | 31.32 |  |
| Poor | 12.70 |  | 24.56 |  |
| Missing | 0.00 |  | 0.36 |  |

*Notes:* One individual can have several disability types. Student t test with unequal variance is used for age, Fisher’s exact tests for other variables. Missing category excluded from the tests.

#### **What if we only used item ratings to infer the ranking (Version 3)?**

Discrepancies between ranking and rating data are expected due to various factors detailed in 2.4. Version 2 (the sensitivity analysis) examine the responses of participants who indicated that all domains were equally important because selecting equal rankings reduced the survey length by two questions and likely lowered the cognitive burden, as ranking tasks can be perceived as more demanding. This introduced a non-preference-related motivation for choosing equal rankings, which justified our targeted analysis of this subgroup.

In theory, there is therefore little reason to conduct a similar check for participants who provided differentiated rankings, as their responses were less likely to be influenced by such survey design factors. Nevertheless, for completeness we use the subjective well-being (SW) items in Table S3 to compute an average rating for the eight life domains, regardless of whether participants declared equal ranking or not. It is worth highlighting that no participant can have strict preferences over the eight domains given that (at most) five levels of importance are available. Generally, someone with strict preferences over the eight domains could still consider all items in the SW to be “very important” and therefore have only one rating (in which case the person would be considered to be indifferent across all domains). Inferring a ranking from the rating data therefore introduces substantial noise.

Table S5 shows the ranked-ordered logit model and corresponding shares as detailed in 2.3. The finding about Work being the least important domain still holds. The Lifelong learning and Participation modules are still among the least important domains (after Work). Finally, Home is still among the most important domains (the most important under Version 3). Choice & Control become the second most important domain when it was generally ranked the fourth important ones. Daily Living becomes only the fifth most important domains when it was generally considered the third most important domains.

Table S5. Ranked-ordered logit model estimates on the relative importance of NDIS Outcomes Framework domains solely based on responses to the items in the SW module (Version 3)

| **NDIS DOMAINS** | **(1)** | **(2)** | **(3)** | | **(4)** | | | **(5)** | | | **(6)** | |  |
| --- | --- | --- | --- | --- | --- | --- | --- | --- | --- | --- | --- | --- | --- |
| **(Ref. Work)** | **Young**  **People** | **Adults** | **Pool** | | **Young**  **People** | | | **Adults** | | | **Pool** | |  |
| **Health & Wellbeing** | 0.857 | 1.310 | 1.210 | | 13.1% | | 13.7% | | 13.6% | | |  |  |
|  | (0.094)** | (0.053)** | (0.047)** | |  | |  | |  | | |  |  |
| **Home** | 1.356 | 1.913 | 1.789 | | 21.6% | | 25.1% | | 24.3% | | |  |  |
|  | (0.107)** | (0.059)** | (0.052)** | |  | |  | |  | | |  |  |
| **Daily living** | 0.698 | 1.253 | 1.131 | | 11.2% | | 13.0% | | 12.6% | | |  |  |
|  | (0.098)** | (0.054)** | (0.047)** | |  | |  | |  | | |  |  |
| **Choice & Control** | 1.119 | 1.474 | 1.394 | | 17.0% | | 16.2% | | 16.4% | | |  |  |
|  | (0.093)** | (0.055)** | (0.048)** | |  | |  | |  | | |  |  |
| **Relationships** | 0.860 | 1.341 | 1.236 | | 13.1% | | 14.1% | | 14.0% | | |  |  |
|  | (0.106)** | (0.054)** | (0.048)** | |  | | |  | | |  | |  |
| **Social, Community** | 0.562 | 0.891 | 0.820 | | 9.8% | | 9.0% | | 9.2% | | |  |  |
| **& Civic participation** | (0.101)** | (0.052)** | (0.046)** | |  | |  | |  | | |  |  |
| **Lifelong learning** | 0.439 | 0.349 | 0.361 | | 8.6% | | 5.2% | | 5.8% | | |  |  |
|  | (0.093)** | (0.038)** | (0.035)** |  | |  | | | |  | | | |
| **N** | 228 | 910 | 1138 | | Work=5.6% | | | 3.7% | | | 4.1% | |  |

Notes: the sample consists of those who completed the SW module and answered at least the first question in the ranking module. Robust standard errors are in parentheses. ** p<0.01, * p<0.05. Columns (1) to (3) shows the results of the regression while columns (4) to (6) displays the corresponding preference shares with those in red the three least important ones and in green the three most important ones.

## **Appendix F. Pooling Age cohorts and statistical differences for the domains**

Table S6. Ranked-ordered logit model estimates on the relative importance of NDIS Outcomes Framework domains

| **NDIS DOMAINS** | **(1)** | **(2)** | **(3)** | **(4)** |
| --- | --- | --- | --- | --- |
| **(Ref. Work)** | **Pool**† | **Pool**‡ | **Pool**† | **Pool**‡ |
| **Health & Wellbeing** | 2.160 | 1.580 | 1.948 | 1.544 |
|  | (0.116)** | (0.057)** | (0.250)** | (0.146)** |
| **Home** | 2.101 | 1.759 | 1.942 | 1.643 |
|  | (0.105)** | (0.041)** | (0.249)** | (0.135)** |
| **Daily living** | 2.009 | 1.671 | 1.723 | 1.618 |
|  | (0.105)** | (0.051)** | (0.230)** | (0.138)** |
| **Choice & Control** | 1.754 | 1.625 | 1.384 | 1.573 |
|  | (0.098)** | (0.054)** | (0.219)** | (0.145)** |
| **Relationships** | 1.732 | 1.668 | 1.578 | 1.558 |
|  | (0.098)** | (0.051)** | (0.207)** | (0.150)** |
| **Social, Community** | 1.108 | 1.245 | 0.773 | 1.279 |
| **& Civic participation** | (0.087)** | (0.071)** | (0.209)** | (0.178)** |
| **Lifelong learning** | 0.314 | 0.566 | 0.489 | 1.127 |
|  | (0.074)** | (0.055)** | (0.193)* | (0.176)** |
| ***Domain interacted with age cohort:*** | | |  |  |
| *Health & Wellbeing |  |  | 0.269 | 0.068 |
|  |  |  | (0.283) | (0.160) |
| *Home |  |  | 0.206 | 0.165 |
|  |  |  | (0.275) | (0.142) |
| *Daily living |  |  | 0.358 | 0.087 |
|  |  |  | (0.259) | (0.149) |
| *Choice & Control |  |  | 0.458 | 0.088 |
|  |  |  | (0.245) | (0.157) |
| *Relationships |  |  | 0.198 | 0.159 |
|  |  |  | (0.235) | (0.160) |
| *Social, Community |  |  | 0.414 | -0.021 |
| & Civic participation |  |  | (0.230) | (0.195) |
| ***Lifelong learning** |  |  | **-0.206** | **-0.656** |
|  |  |  | **(0.209)** | **(0.185)**** |
| **P-value of joint test** |  |  | <0.001 | <0.001 |
| **N** | 403 | 335 | 403 | 335 |

Notes: Robust standard errors are in parentheses. ** p<0.01, * p<0.05. The domain “Work” is the reference domain. † Columns (1) and (3) Participants who ranked the eight domains; ‡ Columns (2) and (4) Imputed values for participants who reported equal ranking on eight domains but reported unequal ratings for related items in the SW module and who will be included in the sensitivity analyses. P-value of joint test is the test of joint significance of all interaction terms between the age cohort dummy (adult or young cohort) and the seven domains.

Table S7. Preference share of NDIS Outcomes Framework domains, Pooled cohort

| **PANEL A - PREFERENCE SHARE (VERSION 1, V1)** | | | | |  |
| --- | --- | --- | --- | --- | --- |
| NDIS DOMAINS | Logit based $p_{j}$† |  | Equal  Importance $\bar{p}$ | Weighted  Average ($P_{j})$ | |
| Health & Wellbeing | 0.211 |  | 0.125 | 0.16 | |
| Home | 0.199 |  | 0.125 | 0.15 | |
| Daily living | 0.181 |  | 0.125 | 0.15 | |
| Choice & Control | 0.140 |  | 0.125 | 0.13 | |
| Relationships | 0.137 |  | 0.125 | 0.13 | |
| Social, Community & Civic participation | 0.074 |  | 0.125 | 0.11 | |
| Lifelong learning | 0.033 |  | 0.125 | 0.09 | |
| Work | 0.024 |  | 0.125 | 0.09 | |
| % of N | 37% |  | 63% |  | |
| **PANEL B - PREFERENCE SHARE (VERSION 2, V2)** | | | | |  |
| NDIS DOMAINS | Logit based $p_{j}$† | Logit based $p_{j}$‡ | Equal  Importance $\bar{p}$ | Weighted  Average ($P_{j})$ | |
| Health & Wellbeing | 0.211 | 0.149 | 0.125 | 0.16 | |
| Home | 0.199 | 0.178 | 0.125 | 0.17 | |
| Daily living | 0.181 | 0.163 | 0.125 | 0.16 | |
| Choice & Control | 0.140 | 0.156 | 0.125 | 0.14 | |
| Relationships | 0.137 | 0.163 | 0.125 | 0.14 | |
| Social, Community & Civic participation | 0.074 | 0.107 | 0.125 | 0.10 | |
| Lifelong learning | 0.033 | 0.054 | 0.125 | 0.07 | |
| Work | 0.024 | 0.031 | 0.125 | 0.06 | |
| % of N | 37% | 31% | 32% |  | |
| Notes: † Participants who ranked the eight domains; ‡ Participants identified as misreporting equal ranking of the eight life domains with imputed ranking values based on the sensitivity analyses. | | | | |  |

1. When NDIS Outcomes Framework domains matched onto two items, we computed the average rating assuming the two items are equally important for that domain. The average was rounded up to the nearest integer. [↑](#footnote-ref-2)
2. With only five possible values for rating (1,2,3,4 or 5), there can be at most five levels for ranking. Most participants only used 3 of the 5 possible ratings. We assumed that those who reported all life aspects as equally important should use less levels and therefore assumed two resulting in the creation of a simple two-level ranking variable. [↑](#footnote-ref-3)
